# Exploration of suicidal thinking in people with multiple sclerosis: an interpretative phenomenological analysis

**DOI:** 10.1080/21642850.2026.2632444

**Published:** 2026-02-18

**Authors:** Ashleigh Roe, Alison Livingstone, Fhionna Moore, Sue Turnbull

**Affiliations:** a School of Health in Social Science, The University of Edinburgh, Edinburgh, UK; b Clinical Neuropsychology, NHS Tayside, Ninewells Hospital, Dundee, UK; c NHS Tayside Child and Adolescent Mental Health Services, Perth, UK

**Keywords:** Multiple sclerosis, suicide, suicidal ideation, interpretative phenomenological analysis, qualitative study

## Abstract

**Introduction:**

Suicidal ideation and rates of suicide are higher within individuals with multiple sclerosis (MS). Despite suicidal ideation often being a precursor to suicide attempts, insufficient research has focused on people living with suicidal thoughts, especially so within MS. The current study aimed to explore the experience of suicidal ideation in individuals with MS.

**Methods:**

Interpretative phenomenological analysis using individual semi-structured interviews was employed to explore the experiences of 8 participants who had reported having experienced suicidal ideation following their MS diagnoses.

**Results:**

Three overarching group experiential themes were identified, with inter-related subthemes. ‘When the strain of living feels impossible’ explored factors including feelings of fading identity, burdensomeness, fear and various losses. ‘Living with mental conflict’ reflected the internal conflict individuals faced in relation to suicidal thinking, both during and in the aftermath. ‘Finding ways to get through’ explored individuals’ internal and external ways of coping with MS and suicidal thinking.

**Discussion:**

The findings reflect emotional distress; ongoing adjustment; and the mechanisms by which suicidal thinking can emerge, particularly at times of diagnosis, relapse and stress. The value of both peer and professional support is highlighted, indicating the importance of addressing mental health in people with MS.

## 1. Introduction

Multiple sclerosis (MS) is a chronic, inflammatory condition of the central nervous system (National Institute for Health and Care Excellence [NICE], 2024) and a leading cause of non-traumatic neurological disability within younger adults (Wallin et al., 2019). There is significant heterogeneity in symptoms between individuals, though all typically experience disabling symptoms, including fatigue (Hadjimichael et al., 2008), pain (Kratz et al., 2011), mood difficulties (Peres et al., 2022), cognitive difficulties (Rao, 1995), mobility impairment and dysfunction of the bladder and bowel (Vaughn et al., 2020). A significant challenge for people with MS is the unpredictable disease trajectory and perceived uncontrollability (Malcomson et al., 2008).

There is a higher occurrence of mental health difficulties in people with MS compared with the general population (Young et al., 2024); including a high prevalence of depression (30.5%) and anxiety (22.1%) in people with MS (Boeschoten et al., 2017), compared to a 16% prevalence of either, in the general population (ONS, 2021). Several psychosocial factors are implicated in mood disorders in people with MS, including hopelessness and helplessness (van der Werf et al., 2003), uncertainty about the future (Lynch et al., 2001), inadequate coping strategies (Goretti et al., 2010), loss of recreational opportunities (Voss et al., 2002), lack of autonomy and high stress levels (Patten et al., 2000).

A meta-review of psychosocial adjustment to MS found illness uncertainty and unpredictability a significant predictor of adjustment and mood (Topcu et al., 2020), peaking at times of diagnosis or relapse (Wilkinson & Nair, 2013). Acceptance (of diagnosis, limitations, and changing identity) was also a prominent predictor of mood and adjustment, and coping with MS (Topcu et al., 2020). Acceptance and uncertainty have been found to be significantly related (Pagnini et al., 2014); the unpredictability of MS makes acceptance of it, and coping, increasingly challenging.

Providing information, offering timely support (Köpke et al., 2014), access to healthcare professionals (Methley et al., 2015) and peer support (Gerritzen et al., 2022) are all factors associated with better adjustment. However, an extensive meta-synthesis of the qualitative literature on the experiences of MS identified a perceived lack of knowledge and insufficient support at the point of diagnosis (Desborough et al., 2020). Further, a thematic analysis, found that feeling let down by healthcare (impersonal communications; under-acknowledgement of emotional wellbeing), alongside disease progression and uncertainty, contributed to feelings of isolation and overwhelm, which, for some, led to considerations of suicide (Hunter et al., 2021). Qualitative research has additionally highlighted the subjective experience of identity change (Barker et al., 2019) and how this can impact on family relationships (Hunter et al., 2021; Strickland et al., 2017); and has identified the profound impact on the everyday lives of people with MS of the experience of loss of control and uncertainty (Gaskill et al., 2011; Nissen et al., 2022).

Suicidal ideation, which frequently precedes suicidal behaviour, has been commonly reported by people with MS (Feinstein, 2002; Pompili et al., 2012). Risk factors associated with suicidal behaviour in MS include depression severity, social isolation, younger age, low income, having progressive MS, higher disability level, the early stage of MS, and premorbid suicidal ideation (Pompili et al., 2012). Meta-analyses have reported an estimated prevalence of suicidal ideation in people with MS of 13% (Kouchaki et al., 2020); and a suicide risk ratio of 1.72 in people with MS compared to the general population, with risk increasing at the time closest to being diagnosed (suicide risk ratio = 2.12) (Shen et al., 2019). A heightened risk has also been identified soon after diagnosis in individuals who are depressed or anxious (Gillespie et al., 2024). Another meta-analysis has found a weighted overall prevalence of suicide attempts within people with MS of 0.04 (d'Andrade et al., 2023). It is important to note that not all suicides in people with MS occur within the context of depressive symptoms (Sadovnick et al., 1991), and suicidal ideation can emerge in those with only mild depressive symptoms, or not meeting psychiatric criteria (Gillespie et al., 2024; Lewis et al., 2017).

There is growing acknowledgement for the necessity to expand the evidence base of research exploring people’s lived experiences of suicidal ideation (Jackson et al., 2020; Jobes et al., 2024; Lees et al., 2016). A thematic analysis in people with MS found that perceived loss of control contributed to suicidal ideation for all sixteen participants (Gaskill et al., 2011). Other themes which emerged included family tension, loneliness, hopelessness, MS symptoms, and loss of perceived masculinity/femininity. The authors suggested that suicidal ideation provides a mechanism of feeling in control. Additionally, distress was found to increase at times of diagnosis or relapse.

The integrated motivational-volitional model of suicidal behaviour (IMVM: O'Connor, 2011; O'Connor & Kirtley, 2018) proposes that suicidality is a result of a complex interplay of risk factors divided into three phases: pre-motivational (background factors, e.g. mood, personality, life events); motivational (the development of suicidal ideation in response to feelings of defeat and entrapment); and volitional (transition from thought to behaviour). The progression between each stage is influenced initially by ‘threat-to-self-moderators’ (e.g. social problem solving, coping skills), then ‘motivational moderators’ (e.g. thwarted belongingness, burdensomeness, future thoughts) and finally ‘volitional moderators’ (e.g. having access to means, capability for suicide and impulsivity. Although quantitative research has identified key pre-motivational risk factors, much less is known about the experience of the motivational stage for those with MS. Qualitative exploration is essential to bridge the gap between statistical risk factors and the personal, often concealed, processes which influence suicidal ideation.

### 1.1. Rationale for the current study

Despite suicidal ideation occurring more frequently than suicide attempts or death (Jobes & Joiner, 2019), insufficient attention has been given to people living with suicidal thoughts. Within MS specifically, little research has explored the experiences of living with suicidal ideation, despite our knowledge of heightened prevalence. Existing research, albeit valuable, arguably does not capture the nuanced, deeply personal experiences and often existential dimensions of suicidality. The current study aimed to address this gap in the literature by taking an interpretative phenomenological (IPA) approach to gain insight and enhance understanding of how suicidal ideation is experienced and made sense of in people with MS.

## 2. Materials and methods

### 2.1. Design

An IPA approach was taken to allow a deep exploration of individuals’ meaning making of the significant and complex life experience of suicidal ideation in people with MS. IPA is grounded in three main theoretical concepts: phenomenology, hermeneutics and idiography. These underpinnings work together to guide IPA in exploring and interpreting the personal, lived experiences of people and their subsequent meaning-making (Smith et al., 2022).

### 2.2. Participants

As recommended for IPA, purposive sampling was used to achieve a small homogenous sample to allow an in-depth understanding of the experience (Smith et al., 2022). A total of eight participants who had experienced suicidal ideation following their diagnosis of MS were recruited through MS nurses; see Tables 1 and 2 for participant characteristics. For the purposes of this study, suicidal ideation was considered to be experiencing thoughts about dying and thinking about actions related to one’s death (Jobes et al., 2024). A total of eleven participants were screened via telephone; although all were eligible, three cancelled their interview. All those who attended for interview were eligible and wished to participate in the study. Four participants identified as male, three as female and one as non-binary. The mean age of participants at interview was 48 (SD: 11.40) and the mean age of MS diagnosis was 29.8 (SD: 7.11). All resided in Scotland (UK) and all but one were married. They were drawn from a range of Scottish Index of Multiple Deprivation (SIMD) deciles (1–9), indicating socioeconomic diversity within the sample. However, there was a greater concentration of participants from lower to mid SIMD areas (5/8 fell within deciles 1–4). The majority of participants had relapsing-remitting MS, and one secondary-progressive. Six participants had a comorbid mental health diagnosis and three had engaged in self-harming or suicidal behaviours in the past.

**Table 1.** Individual participant characteristics, with current MS type and age of MS diagnosis.

Identifier	Age Range	MS Type	Age of Diag.
Harry	60–69	Relapsing-remitting	<20
Alex	30–39	Relapsing-remitting	20–29
Katherine	50–59	Relapsing-remitting	30–39
David	30–39	Relapsing-remitting	20–29
Linda	50–59	Secondary-progressive	30–39
Daniel	40–49	Relapsing-remitting	40–49
Ben	40–49	Relapsing-remitting	20–29
Marie	50–59	Relapsing-remitting	30–39

**Table 2.** Collated participant characteristics.

Characteristic	Participants (*n* = 8)
**Identified Gender**	
Male	*n* = 4
Female	*n* = 3
Non-binary	*n* = 1
**Ethnicity**	
White Scottish	*n* = 6
White British	*n* = 1
White Romanian	*n* = 1
**Scottish Index of Multiple Deprivation Decile**	
Range	1^st^−9^th^
**Employment status**	
Unemployed (inc. due to ill health)	*n* = 3
Retired (inc. due to ill health)	*n* = 2
Employed (Full Time)	*n* = 1
Employed (Part Time)	*n* = 2
**Relationship status**	
Married	*n* = 7
Divorced	*n* = 1
**Mental Health Diagnosis(es)**	
Anxiety and Depression	*n* = 2
Depression	*n* = 3
Emotionally Unstable Personality Disorder	*n* = 1
None	*n* = 2
**Previous suicidal behaviours/attempts**	
None	*n* = 5
Overdose	*n* = 2
Overdose and self-harm via laceration	*n* = 1

Inclusion criteria were: individuals over the age of 18 with a confirmed MS diagnosis (of any type) provided by a neurologist who had reported experiences of suicidal ideation following their diagnosis. They were required to be currently receiving routine National Health Service (NHS) neurology appointments. Exclusion criteria were: a comorbid neurodegenerative disorder; not able to complete interview in English; and being actively suicidal (expressing imminent intent to end their life). Participants were required to be sufficiently physically and mentally able to withstand interviewing conditions and able to provide informed consent.

### 2.3. Procedure

Recruitment took place from August to October 2024 in a single NHS department within Scotland. Potential participants were provided with information about the study in a routine outpatient appointment with their MS nurse. For those expressing interest to participate, with consent, their contact details were passed to the first author. Alternatively, potential participants could make direct contact with the first author via email. Individuals were emailed the participant information sheet and consent forms and a telephone call was arranged with the first author to discuss the study and, for those interested, arrange an in-person interview appointment.

The first author interviewed all participants in an NHS outpatient setting. Written consent was obtained prior to demographic information being sought and semi-structured interviews commencing. Interviews were audio-recorded and lasted between 54 and 100 minutes. The interview schedule included eight open-ended questions to guide discussion; with prompts to encourage elaboration (see appendix for interview schedule).

Due to the sensitive subject area, a brief risk assessment was completed following the interview. Participants were provided with a debrief sheet outlining sources of support. Conversation unrelated to the topic was initiated, to promote grounding. A risk management plan for imminent distress was created in collaboration with liaison psychiatry; however, no concerns arose for any participants.

### 2.4. Experts-by-experience

The first author obtained input from voluntary experts-by-experience from the Research Participation Group of the MS Society. Their involvement aimed to strengthen the quality, acceptability and ethical integrity of the study. It incorporated the voices of individuals affected by MS, and, for some, suicidal ideation, to ensure the focus of the research was grounded in reality. Five experts reviewed all participant facing information documents (participant information sheet, debrief form and the proposed interview schedule), and engaged in telephone discussion or email exchange with the first author to provide feedback and discuss the content.

### 2.5. Ethics

The study was conducted in accordance with the Declaration of Helsinki and had approval, prior to recruitment, from the NHS West of Scotland Research Ethics Committee (reference: 24/WS/0020). All participants provided informed consent.

### 2.6. Data analysis

Data analysis was completed by the first author and reviewed with co-author (ST). Participants’ interview data were transcribed verbatim and anonymised. Analysis adhered to guidance for IPA provided by Smith et al. (2022). This involved immersion in the data via reading and listening to a transcript several times. Line-by-line initial exploratory noting pertained to descriptive, linguistic, and conceptual elements to form experiential statements. Experiential statements for individual participants were analysed to create personal experiential themes (PETs). This interpretative process was repeated for each separate transcript. Here, ‘bracketing’ of previously emerged themes was fundamental, in alignment with idiographic procedures and to promote reflexivity. The dataset was then analysed to create group experiential themes (GETs) which incorporated the holistic experiences of the participants. Convergence and divergence was considered between participants’ accounts, to illustrate both the similarities and patterning of connection, whilst also emphasising the uniqueness of an individuals’ experience (Smith, 2011).

### 2.7. Reflexivity

The first author was attuned to how her professional and academic background in mental health may have influenced her approach to data collection and what was focussed on and attended to during data collection and analysis. She had pre-existing perspectives relating to discrepancies across genders regarding openness to discussing mental health and emotions. This had potential to influence the interviewing approach and lead to exercising more caution in probing with men, impacting the depth of their responses. To mitigate this, assumptions were held consciously in mind during interviews. Concurrently, it was acknowledged that losing a family friend to suicide had the potential to influence how she approached and interpreted participants’ accounts. After each interview reflection, supervision and journaling supported the process of monitoring the impact of personal emotions on interpretations. A sample of the analysis was reviewed by a second author (ST), to allow challenging of interpretations, gain alternative perspectives and verify reliability and rigour of data analysis.

## 3. Results

**Figure 1.**
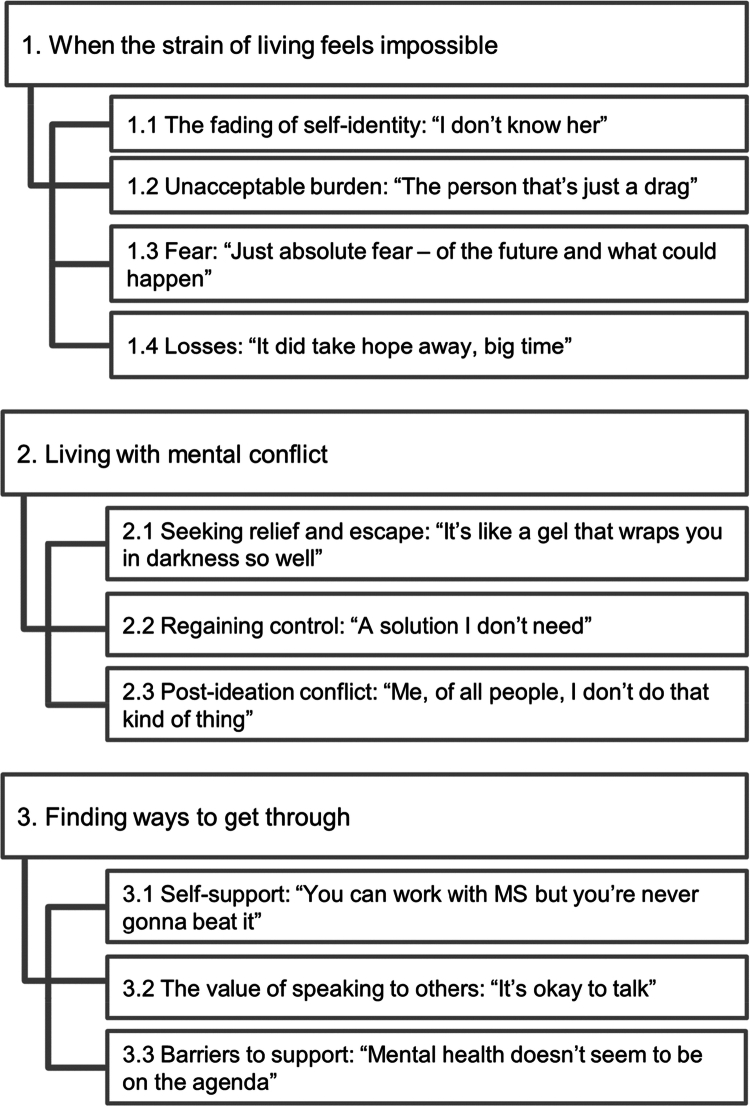
Group experiential themes and subthemes.

This study aimed to explore the lived experiences of suicidal ideation in individuals with MS and the associated meaning making of these experiences. Three overarching group experiential themes (GETs: When the strain of living feels impossible; Living with mental conflict; and Finding ways to get through) each with interrelated subthemes were identified and are summarised in Figure 1.

### 3.1. When the strain of living feels impossible

This explored how participants interpreted the psychological impacts and stressors precipitating the emergence of suicidal thoughts. It included four subthemes: The fading of self-identity: ‘I don’t know her’; Unacceptable burden: ‘The person that’s just a drag’; Fear: ‘Just absolute fear—of the future and what could happen’; and Losses: ‘It did take hope away, big time’.

#### 3.1.1. The fading of self-identity: ‘I don’t know her’

All but one of the participants reflected on how, to some extent, living with MS had resulted in a loss of their sense of self or a shift in how they perceived themselves:

You know, I don’t know her … but this isn’t me, I don’t recognise who this, this person is… I wasn’t bothered what others thought or wasn’t sensitive to letting others down. (Marie)

Marie’s use of third-person language conveyed a sense of self-disconnect which highlighted her struggle in identifying with her current self. There was a disjunction between the person who cared less about others’ opinions, and herself presently, who appears less confident and more emotionally vulnerable. She further elaborated: ‘I feel like a shell of what I was’, which suggested feelings of emptiness and missingness of core elements from her former identity.

Alex described projecting an unappealing version of themselves due to their MS symptoms, an identity which no longer aligned with their true sense of self:

I was putting out, I was showing… something I don’t like, if that makes sense? I’m showing me as not being great, and not at all the way I should be … I’m just, all I can think about is, how do other people see me? (Alex)

The discomfort appeared to be internal but also related to their concern for others witnessing their decline. They perceived themselves to be falling short of their ideal self, creating a sense of inadequacy. Their concern regarding others’ perceptions suggested feelings of being judged which potentially dictated their self-worth. Similarly, for Harry, feelings of being ‘incomplete as a man’ had existed since his teenage years. The diagnosis had robbed him of the opportunity to ‘fully’ form his identity.

A noticeable divergence came from Katherine. Katherine disclosed a history of significant trauma and that ‘MS has been a positive’, because ‘it’s been the easiest’ of the challenges. Whilst other participants expressed negative shifts in identity, Katherine described MS as strengthening her self-perception and being a catalyst to self-growth, affording her the determination to achieve things previously not feel possible. Thus, MS lessened rather than increased psychological strain:

MS sort of built up this fight within me … But it changed my life, honestly, because before that I thought I was useless, totally. I didn’t think I was brainy. I didn’t think I was bright. I went and studied [topic] and I passed it. I can’t believe I passed it - it’s like, how have I done that? Is that me? (Katherine)

She attributed this positive self-development to MS, and interestingly, not to herself. The shift from worthlessness and self-deprecation to disbelief at her achievements, alongside questioning of how *she* achieved this, reflected an ongoing process of redefining and accepting her identity.

#### 3.1.2. Unacceptable burden: ‘the person that’s just a drag’

Burdensomeness was a pertinent theme, with everyone except Daniel and Linda endorsing this. Suicidal thoughts emerged in response to beliefs that participants’ needs were an unacceptable burden to their families, with suicide acting as a solution. Marie viewed herself as ‘the person that’s just a drag’ and expressed: ‘they’d get over it, and at least they could have a fulfilling life without having this burden around their neck’. This reflected that feeling like a hindrance was strong enough to overshadow the devastation her family would face in her absence. Self-sacrifice permeated her interview, suggesting low self-worth and highlighting the emotional strain experienced with burdensomeness. This resonated with Alex and Ben:

Obviously, I thought, if I wasn’t here, they wouldn’t have to help me. They’d be able to focus more on themselves. I had to get my wife to start helping me more, which, I thought, that’s not why she’s here. That’s not what marriage is about. (Ben)

This suggested an internalised sense of failure for Ben. Feelings of guilt and shame emerged from his reflection on what marriage ‘should’ be, as if he had violated this.

#### 3.1.3. Fear: ‘just absolute fear—of the future and what could happen’

Fear was pertinent and presented in different ways for Alex, Ben, Harry, Katherine and Linda, though consistently stemmed from the unknown and lack of control over MS. This occurred at the point of diagnosis for Harry: the lack of information given about MS and his ‘ignorance’ left him ‘thinking the worst’. Similarly, the unpredictability of MS for Ben fuelled fear: ‘Deterioration. You never know when it’s going to happen… the unknown. And that’s what makes, I think, what makes MS scary—you never know, is MS going to kill me?’.

A divergence in experience was noted in Linda, who exhibited acceptance of inescapable decline, referring to a ‘certainty of doom’, with an apparent absence of fear and a desire for assisted dying. Her experience appeared detached from the emotional turmoil faced by the other four, possibly due to the threat element being removed by this way of thinking.

#### 3.1.4. Losses: ‘it did take hope away, big time’

All but Daniel and Katherine conveyed how losses precipitated psychological strain. For some, loss pertained to aspects of their previous lives: abilities, role and purpose, and key features of their identity. In contrast (or additionally), for Harry, Linda and Marie, loss was tied to things that were now unattainable. Thus, losses encompassed both what was lost and what could never be had.

For Marie, her MS diagnosis ‘took away’ her plan to have a child. Increasing resentment built as further sacrifices occurred: ‘One of the hardest things I’ve had to deal with is how much I’d had to give up.. when nothing else fills the void of what you used to be, it’s very difficult to look forward’. Her words implied being ‘robbed’ of options and reflected the magnitude of unwanted changes.

Harry conveyed similar bitterness as to what MS denied him: ‘I missed out on the teenage.. to a great extent, the young man era. I probably blame the MS for what amounted of me—missing out on stuff, you know, a great deal of your younger life’. This conjured a grieving for the loss of experiences during a critical time for identity formation, resulting in feelings of incompletion.

Linda’s narrative encapsulated a shift in her outlook on life in which previous hope, associated with security and autonomy, became unachievable, a loss of possibility:

There was always hope that one day you would win all this money and you could do whatever you wanted and have whatever you wanted... But when, when I started to lose my leg ability, all that hope went. It did take hope away, big time. (Linda)

### 3.2. Living with mental conflict

The second GET encapsulated the cognitive dissonance experienced by participants, both within the process of suicidal ideation and in the aftermath. It included three subthemes: Seeking relief and escape: ‘It’s like a gel that wraps you in darkness so well’; Regaining control: ‘A solution I don’t need’; and Post-ideation conflict: ‘Me, of all people, I don’t do that kind of thing’.

#### 3.2.1. Seeking relief and escape: ‘it’s like a gel that wraps you in darkness so well’

All participants reflected on the underlying function of their suicidal thoughts. A common narrative suggested that suicidal thoughts offered an escape from living with MS and provided a sense of relief though often with a paradoxical quality. Daniel perceived society as demanding contribution to fit in. At times when MS symptoms rendered him unable to, suicidal ideation offered a sense of belonging:

An escape … All of a sudden, it’s like a gel that wraps you in darkness so well and you fit, you fit so perfectly there. And the gel fits perfectly on you and understands you. And makes you feel like oh, fuck, I God damn belong here. It gives you that sort of comfort, that finally you found your place … When you don’t fit for the white gel, you fit perfectly for the black. (Daniel)

Nonetheless, he described this place as ‘comfortable but shit’ and strove to avoid it by giving to others, pushing beyond his own limits, equating his exhaustion and self-sacrifice to a form of suicide, via shortening his life through overexertion. His behaviour reflected an internal conflict, marked by contradictions and potentially influenced by deeply held beliefs formed following significant childhood trauma.

Ben’s account differed; whilst suicidal ideation provided an escape, it was a frightening solitude:

And for them 17 minutes it was just you in the whole world. There was just you and nobody else in your thoughts. Scary, so very, very scary. I did not think about my MS. I did not think about anything or anyone. I just thought about jumping off that bridge, almost like an escape. (Ben)

Ben described a narrowing of his world, feeling unable to look at the bigger picture. That time period represented a rare moment of complete isolation and switching off from considering other people. Contemplating jumping off the bridge offered an instinctual escape, though concurrently, was not a relieving experience.

#### 3.2.2. Regaining control: ‘a solution I don’t need’.

For half of the participants, suicidal ideation provided a sense of regaining control and empowerment at a time they otherwise felt powerless, illustrated by Alex: ‘It’s something I can control, I can control the thoughts. Everything otherwise feels out of control and I just want… something’. The ability to control these thoughts provided fleeting autonomy. The use of ‘something’ highlights just how far beyond their power everything feels and their yearning for this. Simultaneously, a paradox existed that Alex recognised, ‘I usually get scared shitless of them [thoughts]’. This way of thinking appeared to provide a ‘safety net’ for Alex, where they experience control in the absence of intent to act: ‘It’s problem solving a problem that shouldn’t be there. A solution I don’t need’. This contrasted with Linda’s account.

Linda viewed the world as unjustly anti-suicide and wished for assisted dying. She exhibited a considered approach to dying, and a longstanding desire to control her ending:

I never really used the term ‘I want to die’. It’s just the term ‘I don’t want to live like this’. I can’t see, in the last five years, a way out of living like this. Switzerland would be the best plan, the best option, the best end, it would be the best… it would be like planning your own wedding, you know. I just want to plan my end. (Linda)

Whilst she did not outwardly express emotional distress, there was an inferred sense of exhaustion and hopelessness, and desire for control in a situation that feels unbearable. She viewed her thinking as rational and within the interview a sense of isolation emerged from her perception that others’ views do not echo hers, perpetuating a sense of loneliness. Linda referred to other potential options of ending her life, and whilst these are ‘perfectly achievable’, there appeared an element of ambivalence. This was reflected by her acknowledgement of her value: ‘So if I killed myself would I stand aside and look at it and say ‘what a waste’? I probably would, actually, yeah. As much as I am thoroughly miserable living like this, I am still contributing’. This revealed a tension between wanting to escape her pain, and an understanding, potentially reluctantly, that her life holds worth.

#### 3.2.3. Post-ideation conflict: ‘me, of all people, I don’t do that kind of thing’.

Everyone except Alex and Daniel reflected on the aftermath of considering suicide, reflecting feelings of self-criticism, shame, disbelief, and ambivalence. David and Ben appraised their thinking in a critical manner: ‘I don’t understand for the life of me, why I was thinking it. I don’t get it’ (David) and perceived a benefit to him having been a ‘coward’. This statement reflects his challenge of aligning his current self-awareness and perspective, and his previously considered actions. His use of the word ‘coward’ possibly reflected a prior perception of weakness, which has, in turn, become a protective factor he appreciated.

Similarly, Katherine held feelings of embarrassment at having acted on her thoughts, apparently driven by incongruence between her actions and her self-perception. This was following a relapse and commencing steroid medication, which led to mood changes:

I am just so mortified at the fact that, me, I’m just not someone who’d do that. The thing is, like, when I get steroids and that again, I know the same thing could happen. And that scares me. (Katherine)

Further dissonance stemmed from the acknowledgement of the possibility of it re-occurring. She did not feel confident that these feelings would stop the same thing happening and this created fear. Opposingly, whilst Harry reflected on his overdose with emotional disconnect: ‘..it’s almost like somebody else, because I just laugh at it, you know in my head when I think ‘you’ve done that’’; he held no regret. He regarded his overdose as a positive experience, from which he grew and developed a belief ‘there must be something left for me to do in this world’. Thus, he perceived a greater reason for his survival, despite his disbelief at his actions.

### 3.3. Finding ways to get through

The final GET addressed the varying ways participants managed their experience of suicidal ideation and mental health, alongside associated challenges. It included three subthemes: Self-support: ‘You can work with MS but you’re never gonna beat it’; The value of speaking to others; ‘It’s okay to talk’; and Barriers to support: ‘Mental health doesn’t seem to be on the agenda’.

#### 3.3.1. Self-support: ‘you can work with MS but you’re never gonna beat it’

All participants shared strategies they adopted to manage the emotional impact of MS. Some described an internal focus: keeping busy and setting goals to maintain a sense of purpose; humour; or developing an appreciation for the present moment and the goodness in life. Half of the participants described finding value in helping other people. Daniel’s account is multifaceted, set within a background of childhood trauma:

Specifically, when you make decisions in life that are based on emotions, they’re always shit … Why would I feel emotional about the fact that, hey, I’m dying... is it good? I don’t care. (Daniel)

For him, emotions can be detrimental, which is likely to represent a coping mechanism developed over time, in the face of intolerable distress and adverse experiences. Shutting off from emotions serves to protect him; his apparent lack of care toward dying could represent resignation to his situation, or a numbness shielding against emotional weight. Instead, he chooses to ‘focus on other people and to make sure that other people they don’t suffer and be happy’ and ‘I’m keeping myself busy, when you stop focusing on other things, that friend is coming. Hello darkness, my old friend’. Thus, his focus remains external, seeking to avoid suicidal ideation in this way.

Working toward acceptance of MS was reflected in Katherine’s account. This differed from Daniel’s in that she had learned to stop fighting against MS and to live within her remit. She recalled:

I will never forget her words [nurse’s]. And I was the type of person who was not backing down to this, and she said ‘Katherine, you can work with MS but you’re never gonna beat it’ and that stuck in my head 24 years later. (Katherine)

This quote reflects the lasting impact of advice given and how instead Katherine is now working alongside MS.

#### 3.3.2. The value of speaking to others; ‘it’s okay to talk’

Other than Daniel and Katherine, participants discussed peer support; for some, there was a clear benefit of being with people who ‘get it’, whereas for Alex and Linda, peer support had downfalls. There was a consensus with the three male participants endorsing this theme that stigma around males discussing their emotions is shifting. Ben described the value of Andy’s Man Club:

It’s changed the way I think, and I dare say, probably, it’s going to save my life at some point … all these strangers are men, there’s 10-12 men telling me what’s going on in their life - so that’s what’s telling us it’s ok to talk. (Ben)

This conveyed a powerful shift for Ben; he anticipates that suicidal ideation is inevitable in future, but he feels ‘allowed’ to share this with others to support himself. This coincides with a longstanding personal belief that, in response to suffering, he should ‘man up’. He has gained a sense of mutual understanding from these ‘strangers’ resulting in his altered perspective. Similarly, Harry acknowledged the contrast between peer support and receiving redundant reassurance from people who are ‘irrelevant to you’ having not lived the experience.

Alex’s experience of peer support groups differed in that ‘the mental health stuff doesn’t really get touched on at all. I think it’s, well, *I* don’t want to bring the group down… it’s easier to talk about the physical stuff’. This reflected Alex’s own reluctance to discuss emotions due to fear of burdening others with their struggles. In turn, this may lead Alex and others to build perceptions of peer support based on their own avoidance.

Linda provided a distinct take on this subtheme; ‘I haven’t lived for 50 years with the hope that I would be sitting in a group of people with MS’, which reflected frustration at unexpected life circumstances. She perceived no benefit to peer support or value in speaking to others, serving only to remind her of the difficulties she faces.

#### 3.3.3. Barriers to support: ‘mental health doesn’t seem to be on the agenda’

All but Daniel and Katherine’s accounts acknowledged barriers to support, or pitfalls in existing ‘professional’ support, for mental health. David perceived that ‘mental health doesn’t seem to be on the agenda’ for MS and physical symptomatology is the sole focus. Harry corroborated:

There should be a kind of watch placed on your mental health deterioration, especially early on, because not everybody gets stronger by it… there has to be almost like a parallel, a parallel treatment with mental health and MS diagnosis. (Harry)

Harry struggled mentally, early on, due to a perceived lack of knowledge and guidance about MS being provided which heightened distress. All except Harry and David had, or were accessing, NHS or private psychology. Linda and Alex described how therapy had supported in the process toward acceptance of MS. Alex described ‘…everything just kind of clicked and my brain went ‘okay, we’re over this’’. Marie recalled, ‘It was called acceptance and commitment therapy, that I clicked with really well’. However, she acknowledged incongruence between the physical prognosis given and relative mental health support provided:

I keep getting told every time I see a neurologist ‘It’s a progressive disease. It’s only going to get worse’ … I think even if it was a 12-month review, just to know that you are still in the system if you need it because the idea that you have, you know, 12 weeks of psychology and then you’re fixed isn’t real. It doesn’t go away. (Marie)

Being ‘in the system’ appears to provide a sense of security amidst the psychological toll MS can have. Marie’s account advocates for more integrated, holistic approaches to MS care to better meet the needs of this population. Overall, a clear consensus between participants emerged that mental health support is not clearly integrated into MS care or sufficient, nor are interactions with healthcare staff psychologically informed.

## 4. Discussion

Three key themes were identified to support our understanding of the experience of suicidal ideation in people with MS: ‘When the strain of living feels impossible’; ‘Living with mental conflict’; and ‘Finding ways to get through’.

### 4.1. When the strain of living feels impossible

This encompassed participants’ perceptions of their self-identify fading or becoming fragmented, alongside feeling increasingly burdensome to others. Participants’ experiences comprised a sense of fear, related to the unknown and uncontrollability of MS, and an accumulation of losses.

In considering the impact of MS on self-identity, ‘new’ identities formed, which were typically not due to choice. It was apparent that, for some, this was experienced as unfamiliarity with themselves or feelings of incompletion e.g. as a ‘man’ or parent, mirroring observations of identity change by Strickland et al. (2017). Attempts to conceal from others, or deliberately distance from the ‘new’ self were evident. Barker et al., (2019) have proposed that denial and compartmentalisation occur in the early stages of MS with the full impact of identity change experienced through the process of relapses and declining function as MS increasingly becomes part of an identity. They suggest that social support and positive interpersonal interactions may be influential in helping individuals integrate MS into their held identity; and for those who struggle to accept this identity change, adjustment is more difficult, and less peer-support is sought. This was perhaps particularly evident in one participant in the current study, who had a diagnosis of secondary-progressive MS who adamantly avoided other MS sufferers and expressed a wish for assisted suicide.

Perceived burdensomeness was a common experience of participants, in line with previous research (Gaskill et al., 2011; Hunter et al., 2021). The interpersonal theory of suicide (Van Orden et al., 2010) proposes that physical disability leading to the perception of burdening others, especially close family, contributes to an individual appraising suicide as a solution. The strong desire for MS not to impact loved ones in this study was clear. In the context of the IMVM (O'Connor, 2011), burdensomeness acts as a motivational moderator allowing entrapment to translate to suicidal ideation. This was also reflected in the fear participants described surrounding the uncontrollability of the future of what MS may bring. Various losses associated with MS impacted on sense of belonging, goals and aims for the future and, for some, hope. These themes of fear and losses can be understood as motivational-moderators of the IMVM increasing the likelihood of ideation progressing to behaviour. Macintyre et al., (2021) has further proposed that factors such as thwarted belongingness and perceived burdensomeness lead individuals to consider suicide due to goal conflict. Trying to reduce the pain of one, increases the impact of the other; in other words, attempting to reduce burdensomeness might mean reducing their sense of belonging, and vice versa.

Many participants spoke of losses and changes to their identity that resulted in feelings of hopelessness and defeat; findings similar to Barker et al. (2019). Defeat and entrapment are hypothesised to be key drivers in the development of suicidal ideation (O'Connor & Kirtley, 2018), with internal entrapment (trapped by pain linked to internal thoughts and feelings) more likely to lead to suicidal ideation than external entrapment (Owen et al., 2018). One participant described experiencing ‘tunnel vision’ when suicide becomes the only perceived coping mechanism (O'Connor & Nock, 2014).

### 4.2. Living with mental conflict

Cognitive dissonance, marked by inherent internal conflicts, was evident in participants’ narratives. Ambivalence about dying was expressed in that participants did not hold a current wish to be dead, rather a desire for relief and escape from living as they are. Galasiński and Ziółkowska (2024) have proposed that ambivalence serves a protective function, that, in the presence of ambivalence there is no suicide; as long as the internal conflict remains, thought cannot move to action.

Suicidal ideation often represented a means of a semblance of control in participants’ lives, supporting findings by Gaskill et al. (2011), though this ‘escape’ often felt scary and something to actively avoid. Loss of control and uncertainty appears as a prominent concern for people with MS (Gaskill et al., 2011; Nissen et al., 2022); as does the experience of fear (Hunter et al., 2021). Whilst suicidal ideation allowed participants to feel this control, it appeared that creating and having a plan felt protective only in the absence of current intention to implement it. For many, however, acting on thoughts was feasible, given they had access to means (a volitional moderator within IMVM; O'Connor, 2011). That this alone did not always induce action potentially highlights the role ambivalence plays. For those participants who had previously attempted suicide, their narratives suggested that, in line with the IMVM, sufficient volitional moderators can result in transition to suicidal behaviour. Therefore, at times when distress heightens for individuals, healthcare and families should be mindful of the risk of suicide acts increasing.

Participants’ accounts displayed internal conflict in the aftermath of suicidal thoughts or behaviour, in that their thoughts or actions felt uncharacteristic and scary. This reflects previous findings of shock and fragility in men’s accounts of the aftermath of suicide attempts (Richardson et al., 2021). In the current study, shame and discomfort arose for some, potentially due to struggling to hold conflicting emotional states simultaneously; both the wish to remove distress, and a desire to live. This highlights a potential benefit in supporting individuals to develop more dialectical and flexible ways of thinking and navigating their emotional state. There is growing evidence for the value of acceptance and commitment therapy (ACT) in reducing suicidal ideation and behaviours through developing psychological flexibility (Ducasse et al., 2018; Tighe et al., 2018). Dialectical behavioural therapy (DBT) has also been found to be effective in reducing suicidal behaviour, though its impact on reducing suicidal ideation is less convincing (DeCou et al., 2019).

#### 4.3. Finding ways to get through

This GET encapsulated the strategies individuals have developed, alongside support from others. Strategies to manage the impact of MS and, ultimately, avoid suicidal ideation, commonly included suppression or concealing of emotions, using humour, or externally focusing attention and effort; reflecting a prevalence of emotion-focused and avoidant coping mechanisms (Kar et al., 2017) and benefit-finding (Pakenham, 2005). For male participants there was evidence of the suppression of difficult emotions to avoid being perceived as ‘weak’, mirroring the reports of men in Richardson et al. (2021). Though, positively, within both studies male participants felt able to openly share their experiences. Nonetheless, as men are often not as forthcoming, reaching out to them may be required to combat avoidant behaviours adopted in the face of distress (Richardson et al., 2021). Equally, for people of any gender, pre-existing perceptions of burdensomeness may be exacerbated by and act as a barrier to seeking help for mental health.

All participants referred to the value of social connections and interpersonal support, echoing previous findings (Søndergaard et al., 2023). MS specialist nurses often serve as the main healthcare contact for people with MS (Meehan & Doody, 2020), however, the multifaceted demands inherent in this role mean that nursing staff can lack confidence in managing psychological concerns and psychological support is often experienced as an unmet need (Methley et al., 2017; Ponzio et al., 2015). Within the theme of barriers to mental health support, the dynamic between participants (patients) and clinicians was potentially bidirectional. In the absence of clinicians broaching mental health issues, individuals feel less able to raise it themselves. Similarly, the absence of patient-initiated conversations could lead clinicians to avoid the subject further, meaning physical health remains the focus. This was also mirrored, for some individuals, within peer support.

Peer support was experienced as a valuable source of connection for some, though for others it evoked discomfort, either in the confronting nature of seeing future decline as reflected in others, or due to a reluctance to burden peers with their emotional difficulties. Whilst existing research indicates peer-support groups can offer significant benefits (Morris-Bankole & Ho, 2022; Wakefield et al., 2013), their perceived efficacy and acceptability may be influenced by factors including age, gender, symptom severity (McCabe et al., 2015), MS subtype, and level of personal identification with a group (Wakefield et al., 2013). Further, such groups may be detrimental to those with better mental health functioning (Uccelli et al., 2004). Wakefield et al. (2013) suggested a reduced benefit to those with secondary-progressive MS, compared to other subtypes, which they hypothesised as potentially due to disease duration and reduced hope, as reflected in the current study.

An overall sense of inadequate mental health support from healthcare services was conveyed in the present study, which mirrors previous findings, that increased knowledge, training, clearly defined roles and improved access to specialist care is required (Methley et al., 2017). This was from the point of seeking diagnosis, where feeling unheard and invalidated was evident, and the onward process, where poor communication increased distress, echoing findings from previous qualitative enquiries (Hunter et al., 2021; Persson et al., 2023). There was an apparent incongruence between what professionals conveyed regarding the anticipated impact of MS and what was available to support with this, from a mental health perspective.

### 4.4. Strengths and limitations

Limitations of the current study are deserving of acknowledgement. Participants were a small sample of predominantly Scottish individuals with MS, recruited from a single National Health Service department within a specific area of Scotland, all of whom volunteered to take part. Further, they were predominantly married, implying a level of support likely influencing their adjustment. Some individuals may struggle with adherence to healthcare appointments due to factors associated with suicidal ideation. Here, the recruitment process would have limited awareness and opportunity to take part in the study. Speculatively, being able to include those with less adherence to appointments may have been helpful in exploring additional perspectives and experiences. Although volunteer researchers from the MS Society were involved in the initial design stages, due to the unfunded nature of this research, further meaningful paid involvement was not possible. The involvement of experts-by-experience in co-production and collaboration at the analysis stage could have added valuable interpretations and allowed further reflection on the credibility and ethical implications of the findings (Smith et al., 2022).

Positively, there was a relatively even split of genders, reflecting a balance in willingness to discuss such experiences and the participants were drawn from a wide range of socioeconomic backgrounds. Participants’ experiences of suicidal ideation were being explored at a time they felt comfortable to share them. Therefore, it is likely that this filtered the experiences that individuals were able to access and share, which is always a possibility within this type of research. Equally, it would have been unethical to have interviewed people about such experiences at times of instability.

### 4.5. Research implications

Future research could examine how factors such as psychological strain and cognitive dissonance vary across different stages and types of MS. Capturing these dynamics closer to the time of onset or during key transitioning times in MS progression could offer deeper insight into the development of suicidal ideation. A diagnosis of progressive MS has been identified as a risk factor for suicidal behaviour (Pompili et al., 2012) and an exploration of suicidal ideation specifically within progressive MS could add further to the understanding of suicide within this group.

There may be value in research with MS nurses regarding their perspectives in addressing both mental health, but specifically suicidal ideation, in people with MS. Understanding this may support staff training and confidence and enhance support for patients, whereby mental health is not viewed as a lesser priority.

The finding of the experience of ambivalence provides support to Macintyre et al., (2021) who argued that few of the current ideation-to-action frameworks incorporate a consideration of ambivalence as a key concept in suicide, despite its important role and high prevalence (Harris et al., 2010). They proposed that future research may benefit from a framework which provides an in-depth understanding of ambivalence, and the psychological processes involved in the conflict between wanting to live and die. This is especially important given that ambivalence can deter individuals from attempting suicide (Galasiński & Ziółkowska, 2024).

### 4.6. Clinical implications

The current study highlights how crucial it is for health professionals working with individuals with MS to be aware of the possibility of suicidal ideation, particularly at the points of diagnosis, relapse and times of stress. Routinely addressing risk is advised and, particularly, as has been previously suggested, clinicians should screen for suicidal ideation in initial diagnostic sessions (Gillespie et al., 2024). Having courage to broach the topic of suicide in a curious, interested manner is the best way to assess suicidal ideation (Mellentin et al., 2018). The movement in the suicide literature toward ideation-to-action models of suicide, offers beneficial insights into the way we understand and approach risk assessment for suicide, and differentiate between those more likely to move from ideation to enaction. As highlighted by O'Connor and Kirtley (2018) intervention and suicide prevention processes should align with the individual’s current phase within the IMVM, or a similar framework.

Clinicians should be mindful of how all interactions with patients can influence their overall adjustment to MS and ongoing care. The need for accessible information and support at diagnosis is acknowledged within national clinical guidelines (e.g. NICE, 2022), though this can be inadequate (Topcu et al., 2023). A feasibility trial exploring providing resources and coping strategies early in the disease trajectory to enhance overall adjustment acknowledged a tendency for nursing staff to focus on medication and physical health. Insufficient focus on psychological support was reported by participants and delivery of resources by psychology staff in further trials was recommended (Das Nair et al., 2024). Alternatively, this could be supported by up-skilling nursing staff in delivering psychologically informed resources and in identifying those requiring psychological intervention using a stepped-care approach. This may mitigate against mental health worsening, prevent crises occurring, and ultimately reduce service demand.

## Data Availability

Due to the sensitive nature of the research the participants of this study did not give written consent for their qualitative data to be shared publicly, so supporting data is not available.

## Open scholarship

This article has earned the Centre for Open Science badges for Open Materials. The material for the study is the semi-structured interview included as an appendix.

## Appendix: Semi-structured interview schedule

The purpose of this interview is to explore the experiences of suicidal thinking in people with MS. Do you have any questions before we begin?

1. **Can you talk to me about the experience of living with MS and what having MS means for you?** *Prompts: How does it affect you mentally/emotionally/physically/day to day?*
2. **I understand that you have in the past, or still do, experience thoughts about suicide. Please can you tell me about that?** *Prompts: What were you doing/what events led up to you having them? How often have you experienced them?*
3. **Are any of these thoughts related to MS? If so, how?** *Prompts: What makes you think that? What do you remember about the thoughts; can you describe them? Why do you think you have these thoughts?*
4. **Are there other factors not specifically related to MS that contribute to you experiencing suicidal thoughts?** *Prompts: When do these occur? How do your suicidal thoughts related to MS and those not directly related to it compare? How (if at all) does/has MS change/d the experience of these suicidal thoughts?*
5. **How does/did having these thoughts about suicide affect you at the time?** *Prompts: How did it make you feel? In what ways (if at all) were those thoughts helpful/beneficial/serve a purpose? How strong were the feelings? How long did they last?*
6. **Can you tell me how you feel about your experiences of having suicidal thoughts now** (assuming they happened in the past)? *Prompts: What do you think led to this change? (if change is indicated).*
7. **What do you think stops/stopped you acting on your thoughts?** *Prompts: Is there anything in particular that you do/have done that helped you to avoid acting on them? Any particular thoughts you have? Can you talk me through the process?*
8. **How did/do you manage these experiences of having thoughts around suicide?** *Prompts: Does this ever change? What is helpful/unhelpful? What sources of support helped? What do you feel could be helpful?*

Closing questions.

Is there anything else you would like to discuss that has not already been covered, or anything that you think might support our understanding of suicidal thinking in people with MS? What has it been like for you to discuss this topic?

Thank you very much for taking part in this interview.
